# A Few Kind Sentiments

**Published:** 1900-11

**Authors:** 


### "A FEW KIND SENTIMENTS."

THERE is a bizarre sort of a publication issued at East Aurora which sails under the name of *The Philistine* and which is a vehicle for the spleen of a literary harlequin, who, as editor and publisher, makes use of it to advertise himself and to variously attack people dead and alive, and exploit his "beliefs." His name is Elbert Hubbard, and he likes to be called Fra Elbertus.

As a rule the JOURNAL has no liking for digging into the dirty linen of any literary rag shop, yet the October issue of *The Philistine*, in the interests of decency and honor, demands attention. A great many copies have been sent out recently to those who are not subscribers, and physicians have been the chief recipients. The reason for this is apparent for Mr. Hubbard devotes some space to an attack upon Dr. George M. Gould, of Philadelphia, whose works as a writer are well-known, and whose standing as a physician is among the highest.

Dr. Gould was a subscriber to the *Philistine* and he purchased at times books published by Mr. Hubbard. Then, at one time Mr. Hubbard made public in the *Philistine* his "beliefs" concerning christian science and in such manner and wording as to be distinctly offensive to every physician whom he had the good fortune to bear upon his subscription books. Dr. Gould is a forceful and fearless man, dignified and learned, and he wrote Mr. Hubbard a personal letter, returning books by Walt Whitman and Rossetti, in which he said:

> As to "christian science," you have no right to "belief" concerning matters which you have not studied five minutes, and you have less right to voice your "belief." If I ever pretended to have a "belief" about a matter of the most expert inductive science (surgery of the stomach, construction of dynamos, spectrum analysis, etc.,) and to publish that belief when it utterly and absolutely contradicted the opinion of every expert in those sciences, I should hope I would be silenced for my egotism and impertinence. There are one million men in this country who make a life work of the study and cure of disease. You, who have never studied medicine ten minutes, assume to pronounce true what every one of the one million educated physicians can only laugh at as the idiocy of "belief."
>
> Why don't I write you pleasant letters? Because you are all wrong, and have sold your real "beliefs" and independence to the demon of popularity, whom you affect to scorn.

In printing this letter Mr. Hubbard, prefaces it with the statement that it is a private letter and is printed without comment. Then, after violating all the journalistic rules of decency and honor by giving to the reading-public a letter which he considers private—no matter how Dr. Gould looked upon it—Fra Elbertus, let us call him, for his vanity's sake, plunges into a string of abuse of Dr. Gould, because he cancelled his subscription to the *Philistine* "all on account of an innocent little article that expressed a few kind sentiments about our christian science friends," as it is writ by Fra Elbertus. In concluding his attack on Dr. Gould, this man of rag-time literature, who airs his christian science "beliefs" in the *Philistine*, which, by the way, was originally published by a few bright men as a real periodical of protest to stab fraud and puncture the "queer" foibles of the odd side of humanity, and expresses "kind sentiments" concerning as unholy a sect as ever masqueraded and plundered under the cloak of religion, says:

In closing, that I may not be remiss, I wish to thank Dr. Gould for the delicate, though unintentional compliment he pays me in linking my name with that of Walt Whitman and Dante Gabriel Rossetti. To form a trinity with two such men as these has always been quite beyond my fondest hopes. But I am grateful, yes grateful, even tho Rossetti was a victim of "drugs" as Dr. Gould, with allopath accuracy observes. Personally, I do not believe in drugs, and that is my sole offense and cause of this chilly feeling which Dr. Gould entertains toward me. Yet the fact that Rossetti died a victim to the drug-habit will, I trust, on second thought, commend him to the doctor. And possibly, drugs or no drugs, it may not be out of place for me to add that I would rather be wrong and go to hell with Walt Whitman than to be right and play a harp in heaven alongside of Dr. George M. Gould, whose office is at 1631 Locust Street, Philadelphia, Pa.

And he might have added with that unctious, hypocritical grace for which the crowd, which received his "few kind sentiments" is noted. "And here endeth the first lesson."

The JOURNAL is not in accord with Dr. Gould's ideas in many matters of professional dispute, but we are in accord with him thoroughly and heartily in his independence in cancelling his subscription to any publication which defends or even sentimentalises over christian science. It is no discredit to him to suffer attack at the hands of Mr. Hubbard—Fra Elbertus, for vanity's sake; but being a man prominent in the world of letters and medicine, we presume he is at the mercy of such as have attacked him. The only error he has committed is that of writing a personal letter to a Fra Elbertus, without knowing in advance that it would be violated. Mr. Hubbard may prefer christian science support to the friendship of the medical profession. Under the circumstances, he deserves it. It's the worst punishment one could wish him. As to his preferences concerning his location in the hereafter, we have nothing to do. The medical profession labors to save life and to prevent disease; the hereafter is a matter of personal choice and Mr. Hubbard has declared himself. Yet what can we expect from a man who publishes a private letter on one page and on another brutally attacks a dead woman, whose life was beyond reproach? Mr. Elbert Hubbard, of East Aurora, who calls himself Fra Elbertus, does this in the same issue of the *Philistine*, and he damns himself as a ghoul of the most disgraceful type, who digs down into the grave of Mrs. Henry Ward Beecher, and drags her forth from her shroud to ridicule her and the intense love she bore her husband. Is it any wonder that such a man choses hell as his future abode? It is the eternal fitness of things.

---

THE *Galveston Tribune*, at the time of the great storm which nearly submerged that city, had in press a very handsome publication, entitled "Picturesque Galveston." It is a book of something over a 100 pages, printed on 80-pound coated paper, filled with views of this, one of the most beautiful cities in the world.

The editor, Mr. Clarence Ousley, announces that the *Tribune* has tendered the profits of this publication to the Galveston Relief Committee, and under their auspices the book will be sold to the general public at $2.00 a volume.

This is a worthy object and appeals to the better side of human nature. Orders for the book should be addressed to the *Galveston Tribune*, and checks made payable to the same.

---

